# CEVAP journal towards a new phase

**DOI:** 10.1186/1678-9199-19-1

**Published:** 2013-02-27

**Authors:** Benedito Barraviera

**Affiliations:** 1 Journal of Venomous Animals and Toxins including Tropical Diseases, Center for the Study of Venoms and Venomous Animals (CEVAP), São Paulo State University (UNESP), Caixa Postal 577, , Fazenda Experimental Lageado, Rua José Barbosa de Barros, 1780, Botucatu, SP,, 18610-307, Brasil

## Editorial

The *Journal of Venomous Animals and Toxins*[1], ISSN 0104–7930, was established in 1995 as the first electronic Brazilian scientific journal, maintained and edited by the Center for the Study of Venoms and Venomous Animals [2] of UNESP. It has always been written in English and published twice a year, at first distributed on 3.5” diskettes. As the journal took to CD-Rom format in 1998, it was selected to participate in the SciELO Project [3]. In 2003, tropical diseases were included within its scope in order to provide more comprehensive content. It was thereafter entitled *Journal of Venomous Animals and Toxins including Tropical Diseases* (JVATiTD) with ISSN 1678–9199. The journal finally became online-only [4] and was published every four months in 2004. From 2005, JVATiTD began publishing on a quarterly basis and was selected in 2006 to take part in the *Science Citation Index Expanded*™ [5] and Scopus® [6], with inclusion in the EBSCO database [7] in 2010.

JVATiTD has been experiencing significant growth since 2006, in which year 52 articles were published with a rejection rate of 11.86%. The rejection rate has subsequently increased year after year, reaching 41.33% in 2012. The average elapsed time between submission and acceptance from 2007 to 2012 was 135, 139, 133, 88, 86 and 78 days; the respective elapsed time between acceptance and publication was: 196, 143, 170, 132, 95 and 84 days; the overall time between submission and publication was: 331, 282, 303, 220, 181 and 162 days.

Thus, the elapsed time between submission and acceptance is shorter than the one between acceptance and publication. This analysis leads to the conclusion that the speed and visibility of publication have risen significantly in the last five years, given that the current average time between submission and publication is similar to that of journals with higher global impact, that is, only 162 days.

As a consequence of these achievements, there has been a substantial increase in the submission of articles, especially after indexing of the journal in the Journal Citation Reports® (JCR) [8]. JVATiTD received an impact factor of 0.436 in the 2007 JCR which has remained stable since then. Recently, the journal was reclassified by the Brazilian program WebQualis by CAPES [9] in the following strata: A2: Geography; B1: Agrarian Sciences I, Nursing, Engineering III and Interdisciplinary; B2: Biodiversity, Food Science, Veterinary Medicine, Engineering II and IV; B3: Medicine I, II and III, Odontology, Public Health, Pharmacy and Public Health; B4: Biotechnology.

Finally, a partnership with BioMed Central [10] – The Open Access Publisher – will begin in 2013 [11]. It has all come as a consequence of our impact factor stability over the last five years and indexing in the following databases: PubMed [12], PubMed Central [13], SciELO [3], *Science Citation Index Expanded*^*TM*^[5], e-Depot (National Library of the Netherlands digital archive) [14], CrossRef [15], Quertle [16], SCIRUS [17] and Webcite [18].

This partnership with BioMed Central will, beyond the shadow of a doubt, increase our impact factor and, consequently, improve our journal classification worldwide.

## Competing interest

The author declares no conflicts of interest.
